# Chronic Pain among Individuals Experiencing Homelessness and Its Interdependence with Opioid and Other Substance Use and Mental Illness

**DOI:** 10.3390/ijerph19010005

**Published:** 2021-12-21

**Authors:** Marc Vogel, Fiona Choi, Jean N. Westenberg, Maurice Cabanis, Nooshin Nikoo, Mohammadali Nikoo, Stephen W. Hwang, Julian Somers, Christian G. Schütz, Michael Krausz

**Affiliations:** 1 Psychiatric Services Thurgovia, Division of Substance Use Disorders, 8596 Münsterlingen, Switzerland; 2 Center for Addiction Disorder, University of Basel Psychiatric Clinics, 4002 Basel, Switzerland; 3 Department of Psychiatry, Faculty of Medicine, The University of British Columbia, Vancouver, BC V6T 2A1, Canada; fionac@mail.ubc.ca (F.C.); jwestenb@student.ubc.ca (J.N.W.); 19noon@gmail.com (N.N.); mohammadali.nikoo@alumni.ubc.ca (M.N.); christian.schutz@ubc.ca (C.G.S.); m.krausz@mac.com (M.K.); 4 Clinic for Addiction Medicine and Addictive Behavior, Klinikum Stuttgart, 70174 Stuttgart, Germany; m.cabanis@klinikum-stuttgart.de; 5 MAP Centre for Urban Health Solutions, St. Michael’s Hospital, Toronto, ON M5B 1W8, Canada; stephen.hwang@unityhealth.to; 6 Faculty of Health Sciences, Simon Fraser University, Vancouver, BC V5A 1S6, Canada; jsomers@sfu.ca; 7 Centre for Health Evaluation and Outcome Sciences (CHEOS), The University of British Columbia, Vancouver, BC V6Z IY6, Canada; 8 BC Mental Health and Substance Use Services Research Institute, Provincial Health Services Agency, Vancouver, BC V5Z 4H4, Canada

**Keywords:** opioids, stimulants, benzodiazepines, self-medication, risk behavior, homelessness

## Abstract

Chronic pain and substance use disorders are serious conditions that are prevalent among homeless populations. The aim of this study was to examine the association between chronic pain and substance use among individuals experiencing homelessness and mental illness. We analyzed cross-sectional data from two sites of the At Home/Chez Soi study (Vancouver and Toronto) using bivariate statistics and multivariate logistic regression. Substance use and chronic pain parameters were assessed with the Maudsley Addiction Profile and purpose-designed short instruments. The sample comprised 828 participants. Mean age was 42.4 years and 54% reported chronic pain. In bivariate analysis, chronic pain was significantly associated with use of opioids and stimulants, daily substance use, polysubstance use and injecting as route of administration. In multivariate analysis, only daily substance use (OR: 1.46, 95% CI: 1.02–2.09) and injecting (OR: 1.81, 95% CI: 1.08–3.05) remained as significant associated factors, whereas neither use of opioids nor use of stimulants specifically were significantly associated with chronic pain. Among participants with chronic pain, daily substance users (50% vs. 22%, *p* < 0.001) and injectors (66% vs. 24%, *p* < 0.001) were more likely to use non-prescribed medication for pain. Participants with daily substance use were less likely to receive professional treatment (52% vs. 64%, *p* = 0.017) and prescribed pain medication (42% vs. 54%, *p* = 0.023). Our findings suggest an association of chronic pain with patterns related to severity of substance use rather than to specific substance use in homeless persons with mental illness. Interventions aiming at prevention and treatment of chronic pain in this population should consider severity of substance use and associated risk behavior over use of specific substances.

## 1. Introduction

Homelessness is often a chronic condition affecting up to 580,000 individuals on any given night in the United States and about 235,000 Canadians annually [1,2]. Compared to the general population, homeless persons suffer disproportionately from adverse health conditions and chronic diseases [3]. These comprise physical conditions such as infectious disease, or lung, heart and skin problems [4,5,6,7], but also psychiatric disorders. For instance, depression, PTSD and schizophrenia occur more commonly in this population [8].

Substance use is highly prevalent in persons experiencing homelessness. A Canadian study in homeless women found a prevalence of 58% for dual diagnosis (i.e., co-occurring mental illness and substance use disorder) [9]. In the BC Health of the Homeless Survey, a cross-sectional study involving the homeless population of three cities in BC, 55% had concurrent disorders (substance use and mental disorders), and this subsample reported more severe physical and psychological symptoms, along with substantially more difficulties in getting the health care service that they need [10]. For this population, substantial barriers limit access to health care [11,12], which is often delivered in emergency consultations [3]. Using four-year longitudinal data from three Canadian cities in the Health and Housing in Transition Study, individuals with concurrent disorders had significantly higher odds of emergency department use, hospitalization, and primary care visits in the past 12 months after adjusting for potential confounders [13]. Many services still define substance use as an exclusion criterion, further exacerbating this problem [14]. Moreover, problem substance use, psychiatric disorder, and chronic pain have all been documented as risk factors for overdose deaths among homeless individuals [15].

Research on chronic pain in this population is scarce. A study of homeless shelter users in the UK found almost two thirds are affected by chronic pain [16]. We have recently reported a prevalence rate of chronic pain of 58% in the At Home/Chez Soi study in Canadian homeless people with mental illness [17]. Furthermore, we have shown that chronic pain is associated with major depression, PTSD and panic disorder, and that it may contribute to suicidality [17].

The complex relationship of chronic pain with substance use and associated risk behavior in homeless individuals is largely unclear, as it is difficult to determine causality and the specific factors that contribute to the development of chronic pain. Substance use can be a precursor of, self-medication for, or the result of treatment for chronic pain [18]. A study on pain in 483 injection drug users in Vancouver, BC found that history of homelessness was positively associated with self-management of pain through use of illegally obtained drugs [19]. Self-medication may be aggravated because of a higher likelihood of being refused a prescription for pain medication in substance users [20]. In their study on homeless individuals with chronic pain, Hwang et al. found that over half of the participants reported using street drugs for self-medication purposes, but also that participants generally had high rates of comorbid substance use, pain being one of the most identified reasons [11]. By increasing accidents or falls, substance use may contribute to injury, which was the most commonly reported cause for the initial onset of chronic pain in this study. A European study found that mortality of homeless persons was associated with unintentional injury, and history of a substance use disorder and mental health disorder were associated with death due to unintentional injury [21]. The majority (78.9%) of the HIV-positive, indigent participants in the study by Miaskowski et al. had a prescription for pain medication, but they also reported current use of crack cocaine (22.2%), methamphetamines (15.2%), and heroin (5.6%) [22]. This sample had high rates (30.4–55.0%) of lifetime substance use disorder, so current substance use was likely to be more related to pre-existing disorder than current pain.

We use data from the Canadian At Home/Chez Soi study to investigate the association of chronic pain with substance use patterns in homeless with mental illness. Dual disorders were very common in this study and thoroughly assessed with a structured clinical interview (MINI), making it possible to control for their confounding effects [17,23,24]. We hypothesized that the illicit use of opioids and sedatives would be significantly associated with the presence of chronic pain, as participants with chronic pain may use both to self-medicate [25]. Furthermore, we hypothesized that severity of substance use, characterized by daily use, polysubstance (i.e., three or more concurrent substances) use, and injection use would be positively associated with chronic pain.

## 2. Methods

The At Home/Chez Soi study was a Canadian five-center trial conducted from 2009–2013 evaluating the effects of a housing first intervention in homeless persons with mental illness. Participants were recruited from services and institutions for homeless persons such as shelters, drop-in centers, health services and criminal justice programs, as well as community services. Inclusion criteria were adult age, diagnosis of a current mental disorder in the MINI-International Neuropsychiatric Interview [24] (one or more of the following: major depressive disorder, hypomanic or manic episode, PTSD, panic disorder or psychotic disorder), and absolute homelessness or precarious housing. Exclusion criteria were no legal residency in Canada or current participation in an Assertive Community Treatment or Intensive Case Management intervention. Participants were randomized to receive treatment as usual or a housing first intervention, offering subsidized housing and supporting services to homeless individuals. Assessments using standardized interviews administered by trained interviewers took place every three months, but some applied instruments differed by study site. The details of the study design have been described in detail elsewhere [26]. All participants provided informed consent. The ethics boards of all participating centers approved the At Home/Chez Soi study.

The study sites Toronto and Vancouver assessed additional data on substance use as well as chronic pain. For this paper, we use data on substance use in the past month at the 18-months follow-up interview, and chronic pain (defined as persisting pain for at least three months) at the 21-months follow up interview. Self-reported substance use was assessed with the Maudsley Addiction Profile (MAP) [27] in Vancouver and a very similar, purpose-designed instrument in Toronto. Both instruments cover a time period of one month preceding the interview and contain questions on use of several different substances, including whether a substance was used, the number of days it was used, and the route of administration applied. For this paper, use of heroin, illicit methadone or other illicit opioids were merged to the category ‘opioid use’. Use of crack cocaine, powder cocaine, illicit amphetamines and methamphetamines were merged to ‘stimulant use’.

The assessment of chronic pain covered the three months preceding the interview (see Supplementary File S1 for questionnaire). All participants answered the question whether they had suffered from pain for most days in the week during the last three months or more. Further questions on chronic pain were only posed to those participants who confirmed this. These items comprised details on whether participants had sought out pain treatment through a nurse or physician in the prior three months, used prescribed or non-prescribed (street) drugs for pain treatment, and pain interference with general daily activities, sleep, and social interactions. Participants rated the severity of current pain on a visual analog scale (VAS) from 0 (no pain) to 10 (extreme pain).

### Statistical Analysis

All statistical analyses were performed with SPSS (IBM Corp., Armonk, NY, USA). For bivariate analysis of presence of chronic pain, we used chi-squared tests for categorical and Mann-Whitney-U-Test for continuous variables. In order to construct a multivariate logistic regression model with chronic pain as the dependent variable, we used a backward fitting procedure entering all significant variables from bivariate analysis. We tested for interaction and multicollinearity. Variables were kept in the model when *p* was <0.1. Subsequently we used substance use variables from the final logistic regression model for a bivariate analysis of the details on chronic pain. Significance level was set a *p* < 0.05.

## 3. Results

The sample comprised 828 participants from the At Home/Chez Soi study with a mean age of 42.4 years (SD 10.9). A majority of participants reported having chronic pain (N = 448, 54%). Individuals with chronic pain were significantly older than individuals without chronic pain (44.0 years (SD 10.7) vs. 40.5 years (SD 10.9), *p* < 0.001). Use of opioids and stimulants were also significantly more common among individuals with chronic pain. Furthermore, daily substance use, polysubstance use, and injection use were significantly associated with chronic pain. Demographic characteristics for the whole sample and bivariate analysis for presence of chronic pain are displayed in Table 1. Psychiatric characteristics and substance use patterns for the whole sample and bivariate analysis for presence of chronic pain are displayed in Table 2.

The final logistic regression model with presence of chronic pain as dependent variable is displayed in Table 3. Daily use of substances and injection use were kept in the final model, which also included age and the psychiatric diagnostic categories major depressive episode, PTSD and panic disorder. Daily substance use increased the odds for chronic pain by 46%, while injection use of substances nearly doubled the odds. Neither use of opioids nor use of stimulants specifically were significantly associated with chronic pain in multivariate analysis.

Among participants with chronic pain, daily substance users were significantly less likely to have received treatment for pain by a nurse or physician in the prior three months (52% vs. 64%, *p* = 0.017), and to take prescribed (42% vs. 54%, *p* = 0.023) medication for pain. While half of daily substance users indicated taking non-prescribed medication for pain, less than a quarter of participants without daily use reported such behavior (50% vs. 22%, *p* < 0.001). There were no significant differences between those with and those without daily substance use concerning pain interference with general daily activities (73% vs. 75%, *p* = 0.63), sleep (63% vs. 71%, *p* = 0.098) or social interactions (46% vs. 54%, *p* = 0.108), or mean pain severity rated on a VAS (5.15 vs. 5.22, *p* = 0.68).

**Figure 1:**
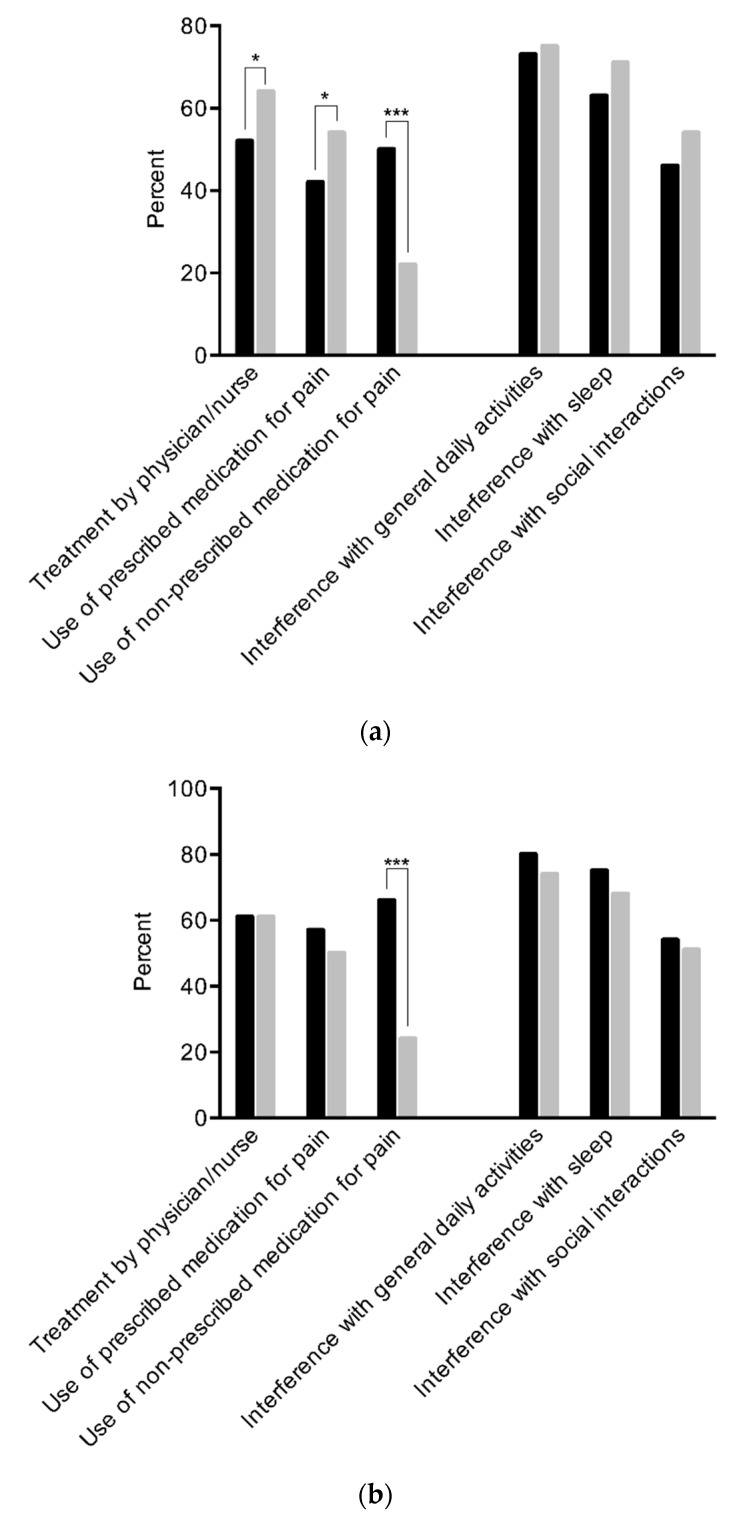
(**a**) Pain-related behavior and pain interference in participants with (black bars) and without (grey bars) daily substance use. Legend: *p*-value from chi^2^-test. * *p* < 0.05, *** *p* < 0.001. (**b**) Pain-related behavior and pain interference in participants with (black bars) and without (grey bars) injection substance use. Legend: *p*-value from chi^2^-test. *** *p* < 0.001.

Two thirds of injection users similarly reported using non-prescribed medications for pain compared to one quarter of participants without injection use (66% vs. 24%, *p* < 0.001). There were no significant differences between those with and those without injection use for having received treatment (61% vs. 61%, *p* = 0.99), taking prescribed medications for the pain (57% vs. 50%, *p* = 0.33), or pain interference with general activities (80% vs. 74%, *p* = 0.31), sleep (75% vs. 68%, *p* = 0.34) or social interactions (54% vs. 51%, *p* = 0.74), or severity of pain (5.70 vs. 5.10, *p* = 0.13). Pain-related behavior and pain interference by daily substance use and injection status are displayed in Figure 1a,b.

## 4. Discussion

This is the first study in homeless persons with mental illness to report on the association of chronic pain with substance use patterns. Our findings suggest an association with parameters indicating severity of substance use (such as daily use and injection use) rather than with use of specific substances.

Concerning the use of specific substances, we found a significant association of illicit opioid use with chronic pain in bivariate analysis. The use of opioids for chronic pain is consistent with the self-medication hypothesis [28,29]. Moreover, a substance use disorder may develop subsequent to the prescription of opioids in the context of chronic pain treatment [30]. However, contrary to our hypothesis, this association did not persist when controlling for age, psychiatric disorders and substance use severity parameters in multivariate analysis. Similarly, a significant bivariate association with illicit stimulant use was no longer found in multivariate analysis.

As suggested by several authors in the field and based on data from various populations, we also expected sedative use to be significantly associated with chronic pain, but this was not the case [25,31]. Homeless individuals with mental illness may be less likely to receive an opioid or sedative prescription compared to other populations. This may be linked to the type of care they receive, especially if provided by emergency systems and not by a continuous care provider (i.e., family physician/GP) due to barriers to care [3,11,12]. Physicians may also be more cautious of the development of a substance use disorder in this often substance-using population and thus be more reluctant to prescribe these substances [11]. Therefore, we were not able to show a pivotal role of the presence of chronic pain for the use of specific substances and vice versa in homeless persons with mental illness.

We found no association between pain and cannabis use, but an association of stimulant use and chronic pain. Both findings do not necessarily support the self-medication hypothesis. Data are limited, but both regular opioid use and regular stimulant use have been demonstrated to reduce pain threshold, with data on stimulants being less strong [32,33].

In the multivariate analysis, clinical indicators of a more severe substance use pattern, such as daily substance use and injection use, but not use of specific drugs, were significantly associated with chronic pain. Chronic pain may be a consequence of substance use and as such more common in people with severe substance use patterns than in those with less severe patterns. Adverse health consequences such as infections or pulmonal and gastrointestinal disease that may lead to chronic pain are more likely to occur in severe substance users (i.e., those with daily use or those with injecting use) than in those with less severe patterns (i.e., those with less than daily use or those with non-injecting use) [34,35]. 

Chronic pain may also have induced daily use in the hope of easing pain with use of the substance. This finding is supported by the use and pain treatment patterns we found. Patients with daily substance use were less likely than those without to receive treatment for their pain by a physician or a nurse. They were also less likely to use prescribed medication for pain and much more likely to use non-prescribed drugs for this intent. Similarly, a larger proportion of injection users indicated the use of non-prescribed drugs for pain compared to people who are not injecting drugs. This finding supports those of Voon et al. who found that substance users were less likely to receive a prescription for pain medication [20]. It is also indicative of the treatment gap in this population, which is partly due to the restrictions on the prescription of pain medications for patients with substance use disorder in the community. These restrictions may have increased since the time of data collection as preventive measures in light of the current overdose crisis. Unfortunately, this may create a situation which can result in an increased rate of illicit use of pain medications and related harms (e.g., overdose) in the absence of adequate alternative multidisciplinary pain programs to cover this gap. Among those with chronic pain, participants with more severe substance use patterns were more likely to bring their substance use into context with chronic pain than their counterparts. Due to our cross-sectional design, we could not establish whether chronic pain leads to more severe substance use or vice versa. However, we did not find an association of severity of pain with daily substance use or injection use. The absence of such a dose–response effect suggests that it may be more probable that patterns related to severe substance use entail the development of chronic pain in this population. 

The type of psychiatric disorders in our sample is very consistent with the comorbidities typically found in dual disorders, which are very common in homeless persons. Both substance use disorders and psychiatric disorders have been associated with chronic pain in the literature. Psychiatric disorders may result as consequences from chronic pain [36], but may also constitute a risk factor for the development of the latter [37]. Furthermore, biological mechanisms and risk factors overlap between chronic pain and psychiatric disorders (e.g., sexual abuse) [38]. It is therefore crucial to control for psychiatric disorders when reporting on the relationship between substance use patterns and chronic pain. This applies not only to homeless persons but also other populations affected by dual diagnosis.

Several limitations have to be considered when interpreting our study results. We only included homeless persons with mental illness in Canada. This may not transfer to homeless persons without mental illness, or to those in other regions with different healthcare systems. Furthermore, substance use was assessed by self-report which may be prone to recall bias or social desirability. Among the strengths of our study are the large sample size of a difficult-to-reach population and the statistical adjustment for psychiatric disorders.

## 5. Conclusions

Our study suggests that among individuals experiencing homelessness and mental illness, chronic pain is a significant problem that relates more to the severity of substance use than to the specific substances. This is a neglected reality in current clinical research. Future studies should investigate whether harm reduction interventions aiming to change harmful substance use patterns can improve chronic pain. The findings have implications for treatment and prevention of chronic pain in this population. The assessment of substance use patterns including type of substance, frequency of use and route of administration should be part of routine care. Interventions aimed at modifying these patterns, specifically including harm reduction measures, should be incorporated into routine care in this population. Overall, expanding treatment access and treatment quality for homeless people with chronic pain is of considerable importance.

## Figures and Tables

**Table 1:** Demographic characteristics and substance use patterns by chronic pain status.

	Total (N = 828) N (%)	Participants with Chronic Pain N (%)	Participants without Chronic Pain N (%)	*p* ^a^
Gender				0.682
Male	572 (69)	310 (69)	262 (69)	
Female	244 (30)	133 (30)	111 (29)	
Other	12 (1)	5 (1)	7 (2)	
Study Site				
Toronto	414 (50)	225 (50)	189 (50)	0.889
Vancouver	414 (50)	223 (50)	191 (50)	
Country of Birth (N = 826)				0.609
Canada	595 (72)	326 (73)	269 (71)	
Outside Canada	231 (28)	122 (27)	109 (29)	
Race/Ethnicity				0.079
Aboriginal	89 (11)	43 (10)	46 (12)	
Ethnoracial ^b^	345 (42)	176 (39)	169 (45)	
White	394 (48)	229 (51)	165 (43)	
Education (N = 824)				0.746
Incomplete high school	429 (52)	234 (53)	195 (52)	
Completed high school	395 (48)	211 (47)	184 (49)	
Marital status (N = 825)				**0.001**
Married/partnered	37 (5)	25 (6)	12 (3)	
Divorced/separated/widowed	216 (26)	137 (31)	97 (21)	
Single/never married	572 (69)	284 (64)	288 (76)	
Primary employment status (N = 825)				0.827
Unemployed	770 (93)	415 (93)	355 (94)	
Employed or self-employed	31 (4)	18 (4)	13 (3)	
Other	24 (3)	14 (3)	10 (3)	
Intervention arm				0.423
Treatment as usual	326 (39)	182 (41)	144 (38)	
Housing first	502 (61)	266 (59)	236 (62)	
Wartime service (N = 819)	39 (5)	27 (6)	12 (3)	0.058

^a^*p*-value from chi^2^-Test; significant *p*-values in bold ^b^ Ethno-racial category includes black, East Asian, Indian Caribbean, Latin American, Middle Eastern, South Asian, Southeast Asian, and mixed ethnicity.

**Table 2:** Psychiatric characteristics and substance use patterns by chronic pain status.

	Total (N = 828) N (%)	Participants with Chronic Pain N (%)	Participants without Chronic Pain N (%)	*p* ^a^
Major Depressive Episode ^b^	319 (39)	205 (46)	114 (30)	<0.001
Manic or Hypomanic Episode ^b^	131 (16)	82 (18)	49 (13)	0.034
Post-traumatic Stress Disorder ^b^ (N = 827)	214 (26)	139 (31)	75 (20)	<0.001
Panic Disorder ^b^	148 (18)	104 (23)	44 (12)	<0.001
Mood Disorder with Psychotic Features ^b^ (N = 827)	158 (19)	96 (22)	62 (16)	0.060
Psychotic Disorder ^b^	365 (44)	174 (39)	191 (50)	0.001
Any substance use (N = 806)	535 (66)	301 (69)	234 (63)	0.067
Alcohol use	367 (44)	204 (46)	163 (43)	0.446
Opioid ^c^ use (N = 818)	89 (11)	57 (13)	32 (9)	0.047
Sedative use (N = 823)	13 (2)	8 (2)	5 (1)	0.580
Stimulant use ^d^ (N = 817)	234 (29)	141 (32)	93 (25)	0.025
THC use (N = 827)	290 (35)	163 (36)	127 (34)	0.388
Daily use of any substance (N = 819)	200 (24)	127 (29)	73 (19)	0.002
Polysubstance (≥3) use (N = 806)	118 (15)	77 (18)	41 (11)	0.008
Injection use (N = 818)	82 (10)	56 (13)	26 (7)	0.007

^a^*p*-value from chi^2^-Test; significant *p*-values in bold ^b^ at baseline. ^c^ includes heroin and prescription opioids ^d^ includes crack cocaine, powder cocaine, amphetamines, methamphetamine.

**Table 3:** Logistic regression model with chronic pain as dependent variable.

	Odds Ratio	95% CI		*p*-Value	S.E.	Wald	Df
		Lower	Upper				
Age	1.038	1.023	1.052	<0.001	0.007	27.924	1
Major depressive episode	1.545	1.125	2.122	0.007	0.162	7.225	1
Post-Traumatic Stress Disorder	1.623	1.132	2.325	0.008	0.183	6.957	1
Panic disorder	1.756	1.161	2.656	0.008	0.211	7.108	1
Daily substance use	1.458	1.020	2.085	0.039	0.182	4.274	1
Injection use	1.810	1.076	3.047	0.025	0.266	4.994	1

Legend: CI = confidence interval, Df = degrees of freedom, S.E. = standard error.

## Data Availability

For data availability, please contact the authors.

## Supplementary Materials

The following are available online at https://www.mdpi.com/article/10.3390/ijerph19010005/s1. File S1: Acute and Chronic Pain.
