# Supplementary material for: Chronic Pain among Individuals Experiencing Homelessness and Its Interdependence with Opioid and Other Substance Use and Mental Illness
Source: Int J Environ Res Public Health. 2021 Dec 21;19(1):5. doi: 10.3390/ijerph19010005 (PMC8751035; doi:10.3390/ijerph19010005)
Supplement: Supplementary file 1 [file ijerph-19-00005-s001.zip › ijerph-1482246-supplementary.pdf]

Acute and Chronic Pain

ID ______ Y 2 0 __ M __ D __

The next questionnaires will ask about chronic and acute pain that you've experienced.

1. Have you suffered from pain for most days in the week during the last three months or even longer?

   If yes, go to 1a. If no, skip the rest of questionnaire.

   ◯ Yes ◯ No

1.a If yes, please indicate the area(s) of pain:

- ☐ Head/Neck
- ☐ Limbs(Arms/Legs)
- ☐ Back
- ☐ Joints
- ☐ Gastrointestinal
- ☐ Face
- ☐ Oral/Teeth
- ☐ Feet
- ☐ Other

1.b If other, specify:

____

2. Have you seen a physician or a nurse for the treatment of this specific pain in the last 3 months?

   ◯ Yes ◯ No

3. Did you take prescribed drugs (prescribed for you by a doctor or nurse) for the pain?

   ◯ Yes ◯ No

4. Did you take non-prescribed drugs (street drugs) for the pain?

   ◯ Yes ◯ No

5. Does the pain interfere with your general daily activities?

   ◯ Yes ◯ No

5.a Does the pain interfere with your sleep?

   ◯ Yes ◯ No

5.b Does the pain interfere with your interactions with other people?

   ◯ Yes ◯ No

Please rate your current pain today. Mark a 'X' on the line below how intense your pain today is.

Use the visual VAS scale with the participant.

6. VAS Score

   __ __

© HealthDiary Inc
